# Optimization of Process Parameters for Laser Cladding of AlCoCrFeNi High-Entropy Alloy Coating Based on the Taguchi-Grey Relational Analysis

**DOI:** 10.3390/ma18194463

**Published:** 2025-09-25

**Authors:** Andi Huang, Yilong Liu, Jingang Liu, Shiping Yang, Jinghao Huang

**Affiliations:** 1 School of Mechanical Engineering and Mechanics, Xiangtan University, Xiangtan 411105, China; andy_h_china@foxmail.com (A.H.); jingangl2025@163.com (J.L.); 2 School of Aeronautical Engineering, Hunan Automotive Engineering Vocational University, Zhuzhou 412001, China; liuyilong@stu.usc.edu.cn; 3 School of Mechanical Engineering, University of South China, Hengyang 421001, China

**Keywords:** AlCoCrFeNi high entropy alloy, laser cladding, Taguchi method, grey relational analysis, coating performance

## Abstract

Aircraft engine turbine discs operate under extreme conditions that limit their service life. Laser cladding of AlCoCrFeNi HEA coatings presents a viable solution to enhance their durability. This study optimizes the laser cladding process parameters—specifically, laser power, scanning speed, and powder feed rate—using the Taguchi method in conjunction with grey relational analysis. The optimal parameter set (1450 W, 480 mm/min, 4 r/min) resulted in a coating with a width of 2.93 mm, a height of 1.20 mm, a dilution rate of 22.6%, and a hardness of 532 HV. The optimized process significantly improved hardness by approximately 15% while reducing dilution and elemental segregation in comparison to the initial parameters. This research illustrates the effectiveness of multi-objective optimization in enhancing coating performance, providing a practical approach for the surface strengthening of critical components, such as turbine discs in aircraft engines, under extreme conditions.

## 1. Introduction

With the rapid advancement of aerospace technology, modern aircraft engines impose increasingly stringent performance requirements on their hot-end components [1,2,3]. Turbine discs, which are critical components that connect turbine blades to the main shaft, experience direct erosion from high-temperature combustion gases while operating under extreme conditions characterized by high loads, elevated speeds, and severe temperature gradients [4,5,6]. Furthermore, these components must endure friction, wear, thermal fatigue, and oxidative corrosion throughout extended service periods, presenting significant challenges to their structural integrity and operational lifespan [7,8,9].

Currently, conventional high-temperature alloys—predominantly nickel-based superalloys (such as Inconel 718 and Waspaloy) and cobalt-based alloys (including Haynes 25 and the Stellite series)—are nearing their theoretical performance thresholds [10]. While these materials remain extensively utilized due to their established reliability and proven track record, their limited potential for further enhancement presents significant challenges in meeting the demanding requirements of next-generation engines, which necessitate higher thrust-to-weight ratios, extended service lifespans, and improved thermal efficiency.

Surface engineering technologies have emerged as promising solutions to address material limitations, with recent advancements showcasing significant enhancements in both mechanical and corrosion performance. Among the various surface modification techniques, laser cladding stands out due to its unique advantages compared to alternative methods. Unlike selective laser melting, which is primarily geared towards additive manufacturing applications, laser cladding offers exceptional control over dilution, making it particularly suitable for surface modification. Yuan et al. demonstrated that high-speed laser cladding resulted in superior microstructural refinement compared to conventional methods [11]. Wang et al. highlighted the complex interdependencies of parameters that critically affect coating quality [12]. Recent technological innovations, such as ultrasonic vibration-assisted processing and hybrid laser-induction techniques, have further underscored the remarkable versatility of laser cladding across a range of industrial applications [13].

High Entropy Alloys (HEAs) represent a paradigm-shifting class of materials characterized by the presence of multiple principal elements in near-equiatomic proportions [14]. This distinctive compositional approach results in four fundamental effects: high mixing entropy that stabilizes simple solid solution phases [15], sluggish diffusion kinetics that enhance thermal stability [16], severe lattice distortion that provides solid solution strengthening [17], and synergistic cocktail effects that enable precise property tailoring [18]. Collectively, these underlying mechanisms contribute to exceptionally high-temperature mechanical properties, superior corrosion resistance, and remarkable thermal stability [19,20].

The AlCoCrFeNi alloy system demonstrates exceptional potential for high-temperature coating applications. Lu et al. successfully developed oxide-dispersion-strengthened AlCoCrFeNiY coatings that exhibit superior oxidation and spallation resistance through AC-HVAF thermal spray deposition [21]. Liu et al. investigated the microstructure and high-temperature wear behaviour of in situ TiC reinforced AlCoCrFeNi-based composite coatings fabricated by laser cladding, highlighting their enhanced wear resistance at elevated temperatures [22]. Odabas et al. evaluated the high-temperature oxidation resistance of AlCoCrFeNiZr high-entropy alloy coatings at 1000 °C and 1100 °C, revealing valuable insights into their performance under extreme thermal conditions [23]. Joseph et al. studied the sliding wear behaviour of CoCrFeMnNi and AlxCoCrFeNi alloys at elevated temperatures, showing significant differences in wear resistance and thermal stability [24]. Zhang et al. explored the hot corrosion resistance of AlCoCrFeNi2.1 coatings at 900 °C, emphasizing their potential for high-temperature applications in corrosive environments [25]. While these studies confirm the substantial potential of AlCoCrFeNi systems, there remains a need to systematically optimize laser cladding process parameters, particularly in the context of laser-based technologies.

To address this, effective process optimization requires the adoption of advanced methodological approaches. The Taguchi method enables efficient parameter optimization through minimal experimental iterations while effectively considering complex parameter interactions [26]. Grey Relational Analysis (GRA) offers a robust mathematical framework for multi-objective optimization by consolidating multiple performance characteristics into unified relational grades [27]. Despite the demonstrated success of the integrated Taguchi-GRA methodology in optimizing laser cladding across various material systems, its application to AlCoCrFeNi high-entropy alloys remains largely unexplored.

This investigation seeks to address the identified limitations by systematically optimizing the laser cladding parameters for AlCoCrFeNi coatings on turbine disc substrates. By employing Taguchi orthogonal experimental design alongside Grey Relational Analysis, this study thoroughly evaluates coating quality across several criteria, including geometric parameters (coating width, height, and dilution rate), mechanical properties (microhardness), and compositional stability (elemental deviation). The primary novelty of this work lies in its integrated optimization approach, which simultaneously addresses multiple quality aspects, thereby offering a comprehensive solution for enhancing the surface strength of aerospace components.

## 2. Materials and Methods

### 2.1. Experimental Materials and Equipment

**Figure 1:**
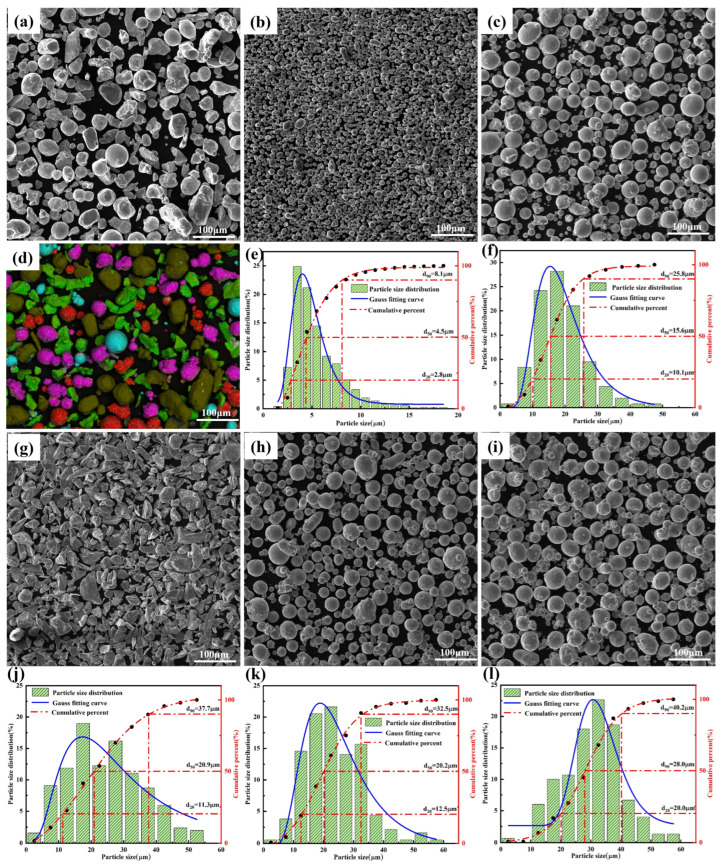
SEM images of mixed AlCoCrFeNi powder and five original powders, as well as powder size and distribution range (**a**,**d**) AlCoCrFeNi and EDS spectrum, (**b**,**e**) Al powder and size distribution range, (**c**,**f**) Co powder and size distribution range, (**g**,**j**) Cr powder and size distribution range, (**h**,**k**) Fe powder and size distribution range, (**i**,**l**) Ni powder and size distribution range.

The elemental powders utilized for laser cladding consist of aluminum (Al:20 at%), cobalt (Co:20 at%), chromium (Cr:20 at%), iron (Fe:20 at%), and nickel (Ni:20 at%), as detailed in Table 1. These powders were mixed in equal atomic ratios using a YXQM-4L (Miqi MITR Instrument Co., Ltd., Changsha, China) planetary ball mill operated at 350 rpm for a duration of 5 h. The ball-to-powder mass ratio was maintained at 3:1, employing stainless steel balls with a diameter of 5 mm. Prior to the laser cladding process, the mixed powders were subjected to a vacuum drying process at 60 °C for 3 h to eliminate moisture. Figure 1 presents scanning electron microscope (SEM) images of the ‘raw powder’ and ‘mixed AlCoCrFeNi powder’. For the substrate of the sample cladding, commercially available 10 mm thick 430 stainless steel sheets were cut into dimensions of 100 mm × 20 mm. The detailed elemental composition of the substrate is provided in Table 2.

**Figure 2:**
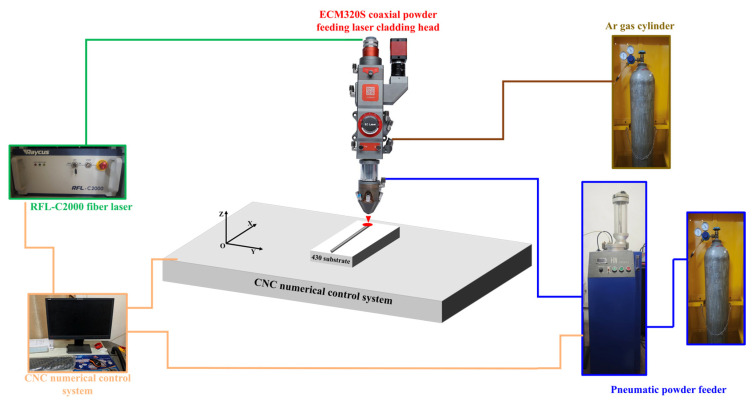
Schematic diagram of laser cladding AlCoCrFeNi HEA coating.

Laser cladding experiments were performed utilizing a laser cladding system that comprised a laser generator (RFL-C2000X, Wuhan, China), an optical fibre (75 µm diameter), an inert gas (Ar) delivery system, a powder delivery system (HW-05SF, Wuhan, China), and a computer numerically controlled three-axis worktable. The diameter of the laser beam spot was 3 mm, with a maximum power output of 2000 W. Figure 2 illustrates a schematic diagram of the laser cladding process for AlCoCrFeNi HEA coatings. Argon was employed as both the shielding gas and the carrier gas for powder delivery.

### 2.2. Experimental Design

This study employed a systematic experimental design and a multi-objective optimization approach to optimize the parameters of the laser cladding process. Initially, based on preliminary experimental results, the feasible domain of the key process parameters was identified. Laser power (P), scanning speed (V), and powder feed rate (F) were selected as variables in a three-factor, five-level experimental design. Through orthogonal experiments, a quantitative relationship model between the process parameters and the geometric properties of the cladding layer was established. As illustrated in Table 3, the experimental matrix encompassed the parameter space for power (1000–1600 W), speed (480–720 mm/min), and powder feed rate (3–5 r/min).

In the parameter optimization stage, a multi-objective optimization system is constructed utilizing the Taguchi method. The specific implementation process consists of the following steps: (1) determining the feasible domain of process parameters through preliminary experiments; (2) conducting single-pass cladding experiments based on the L25 orthogonal table; (3) employing signal-to-noise ratio (S/N) analysis and variance analysis (ANOVA) to quantify the contributions of each parameter to cladding quality; (4) establishing a comprehensive evaluation system that includes multiple index constraints, such as dilution rate, element deviation, and hardness, and obtaining the optimal parameter combination through multi-objective decision-making; and (5) performing verification experiments to confirm the optimization effects. The optimal parameter combination ultimately obtained serves as a foundation for the high-quality preparation of multi-layer and multi-pass composite coatings.

During the coating characterization stage, a multi-scale analysis method was employed to systematically evaluate the performance of the cladding layer under optimized parameters. Macroscopic morphology was observed using a metallographic microscope, while microstructure analysis was conducted with a scanning electron microscope (SEM) (Czech TESCAN model MIRA4, Brno-Kohoutovice, Czech Republic). Additionally, mechanical properties testing was performed using a microhardness tester.

This study uses the Taguchi method to design orthogonal experiments. Table 3 lists three parameters and their corresponding values, with a total of five levels. Table 4 lists the specific conditions of the parameter combinations.

### 2.3. Sample Characterization and Performance Analysis

Cross-sectional observation samples were obtained from single-pass cladding specimens using wire-cut electro-discharge machining (EDM). The samples were taken along the longitudinal midline of the cladding pass. They were processed with a graded grinding technique, sequentially polishing with SiC sandpaper of grits 400, 800, 1200, 2000, 2500, and 3000. Surface contaminants were removed through ultrasonic cleaning in ethanol for 10 min. To reveal the microstructural characteristics of the cladding layer, the samples were selectively etched using aqua regia (a mixture of concentrated hydrochloric acid and concentrated nitric acid in a 3:1 volume ratio) for 15 s. The cross-sectional morphology images of the cladding layer were obtained using a Leica DM2700M optical microscope (Leica Microsystems, Wetzlar, Germany). The geometric characteristics of the cladding layer, including melt height, width, and depth, were quantitatively measured with an accuracy of ±2 μm using ImageJ image analysis software (Image-Pro Plus 6.0).

**Figure 3:**
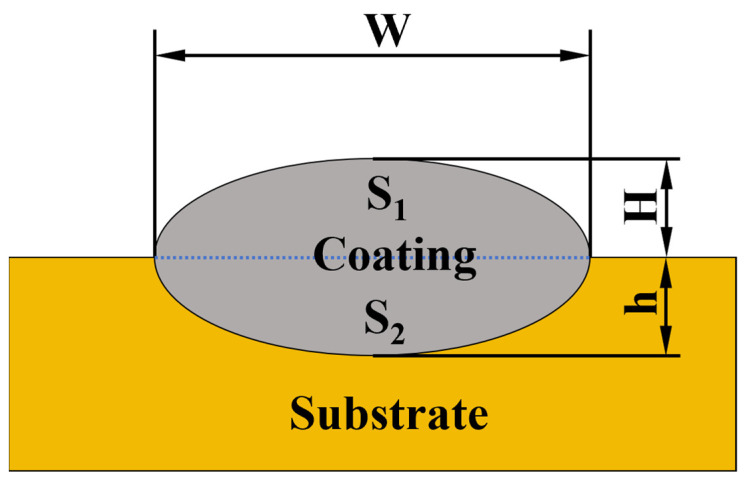
Geometric characteristics of a single laser cladding pass.

To accurately evaluate the influence of the substrate on the composition of the cladding layer, a dual model is employed to calculate the dilution rate *η*. The definitions of the geometric parameters are illustrated in Figure 3, and cross-validation is conducted using both the area method (Equation (1)) and the geometric parameter method (Equation (2)).

(1) $$\eta =\frac{h}{H+h}\times 100\%$$

(2) $$\eta =\frac{S_{2}}{S_{1}+S_{2}}\times 100\%$$

Let S_1_ represent the cross-sectional area of the cladding layer (mm^2^), S_2_ denote the cross-sectional area of the substrate melt zone (mm^2^), *H* signify the cladding height (mm), and *h* indicate the penetration depth (mm). The final dilution rate is calculated as the arithmetic mean of the two methods to minimize systematic errors.

Energy dispersive spectrometer scanning (EDS) (OXFORD XPLORE 30, Oxfordshire, UK) mapping was employed to quantitatively analyze the distribution of major elements across the upper, middle, and lower regions of the cladding layer’s cross-section. For statistical analysis, three representative fields of view (200 × 200 μm^2^) were selected from each region, and the element deviation rate (α) (Equation (3)) was calculated.

(3) $$\alpha_{i}(\%)=\frac{C_{i,measured}}{C_{i,nominal}}\times 100$$

where C*_i,measured_* is the measured atomic percentage of element *i* in the cladding layer, and C*_i,nominal_* is the nominal atomic percentage in the equiatomic AlCoCrFeNi alloy (20 at.% for each element).

Microhardness (φ) measurements were conducted using an HVS-1000 (Shanghai Caikang Optical Instrument Co., Ltd., Shanghai, China) Vickers hardness tester with a test load of 200 gf and a hold time of 10 s. Measurements were taken at intervals of 100 μm perpendicular to the substrate, commencing at 50 μm below the top of the cladding layer. Three sets of parallel data were collected at each depth, with the final hardness value being the average of the nine valid data sets.

## 3. Results

### 3.1. Empirical Statistical Models

#### 3.1.1. Trajectory Morphology

**Figure 4:**
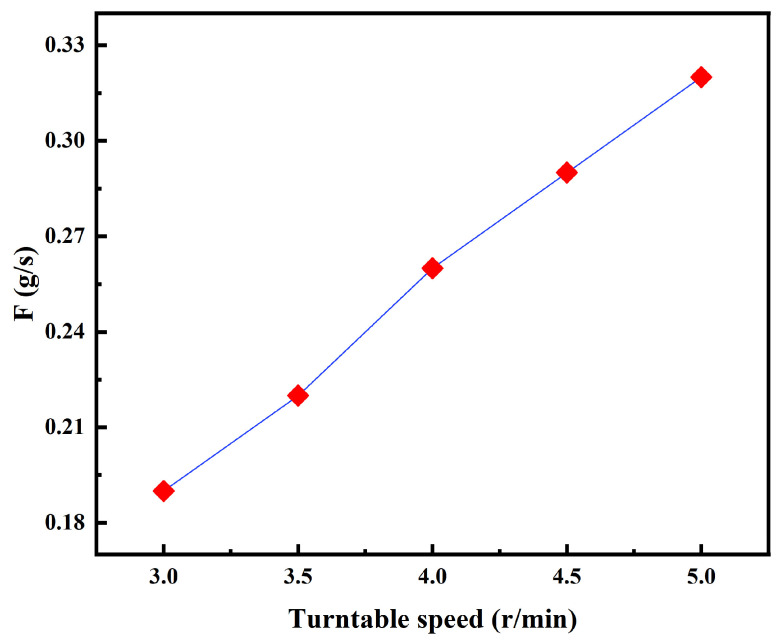
Conversion of powder feeding rate.

**Figure 5:**
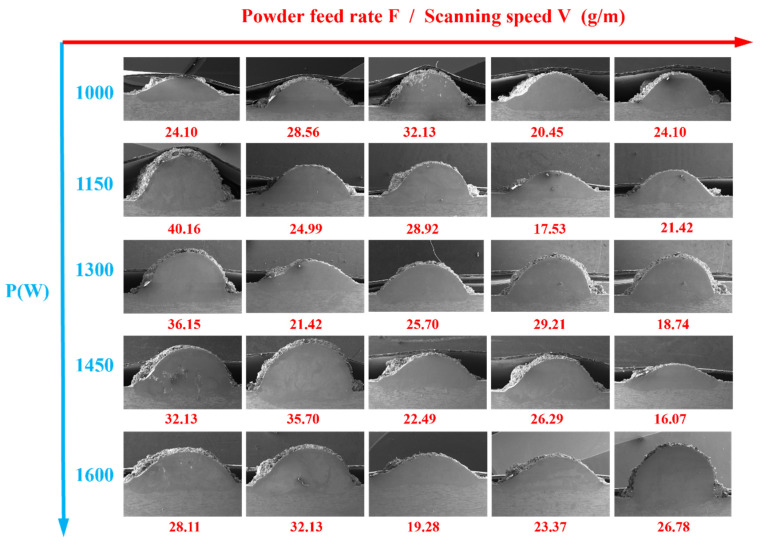
Cross-sectional morphology of single-pass cladding layer under different laser power (P) and powder feed rate-scanning speed ratio (F/V).

The conversion diagram of the powder disc speed and the powder feeding rate is shown in Figure 4. Figure 5 illustrates the cross-sectional morphology of single-pass cladding layers at various laser power settings (P) and powder feed rate-to-scan speed ratios (F/V). All cladding layers display a semicircular profile, which can be attributed to the effects of surface tension during the solidification of the molten pool. The figure further demonstrates that strong convection within the molten pool facilitates a uniform distribution of metal particles during solidification. This visualization effectively conveys the impact of process parameters on the geometric characteristics of the cladding layers.

The height of the cladding (H) exhibits a significant positive correlation with the feed rate to velocity (F/V) ratio. Additionally, laser power (P) positively influences H. Experimental results indicate that porosity defects are more likely to occur in the cladding layer at elevated F/V ratios. This phenomenon is primarily attributed to the extended solidification time associated with thicker cladding layers, which hinders the escape of gases generated by metal oxidation from the liquid metal. The measured data for the geometric parameters of a single-pass cladding layer are presented in Table 5. A systematic statistical analysis will be performed to elucidate the mechanisms by which process parameters influence these outcomes.

#### 3.1.2. Clad Width

**Figure 6:**
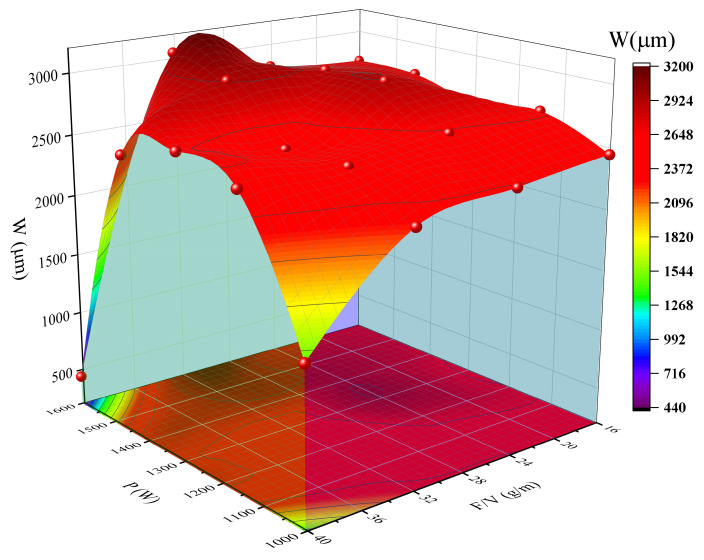
Surface plot of width vs. laser power, powder feeding rate/scanning speed (F/V).

As illustrated in Figure 6, the formation mechanism of the cladding width (W) demonstrates a significant positive correlation with the laser power (P). The experimental data indicate that the power range can be categorized into two distinct domains: in the low-power range (P = 1000–1150 W), the average W is 2428.26 μm, with a fluctuation range of 2177.84–2600.46 μm and a coefficient of variation (CV) of 5.8%. This reflects the instability of the melt pool spread due to insufficient energy supply. Conversely, when the power is increased to the medium-to-high power range (P = 1300–1450 W), the average W rises significantly to 2784.59 μm (CV = 3.2%), indicating a stabilization of the lateral flow of the melt pool. For instance, at experimental point 16 (P = 1450 W, W = 2931.05 μm), a 34.6% increase in power corresponds to a 34.6% increase in width. The Pearson correlation coefficient (R) of 0.82 further substantiates the dominant influence of power on width.

The control of cladding width by the powder feed rate/scanning speed (F/V) exhibits power-dependent characteristics. In the low-power range (P ≤ 1150 W), an increase in F/V inhibits the lateral expansion of the melt pool. For instance, at P = 1000 W, increasing F/V from 24.10 g/m to 32.13 g/m results in an 11.5% decrease in W. This phenomenon is attributed to the absorption and dissipation of laser energy by excess unmelted powder. Conversely, in the high-power range (P ≥ 1300 W), F/V and P exhibit a synergistic effect. For example, at P = 1450 W, increasing F/V from 32.13 g/m to 35.70 g/m leads to only a 5.5% decrease in W. This observation indicates that higher power can partially compensate for the cooling effect of increased powder feed on the melt pool. Notably, experimental point No. 12 (P = 1300 W, F/V = 21.42 g/m, W = 2941.28 μm) demonstrates that high power can still achieve ultra-wide cladding under low powder feeding conditions, further corroborating the dominance of power parameters.

Through parameter combination optimization analysis, it was determined that selecting a process window of P = 1300–1450 W with F/V = 28–32 g/m results in a stable cladding width of 2500–2900 μm. Additionally, it is advisable to avoid the non-steady-state forming ranges associated with low power and high powder feeding (P ≤ 1150 W, F/V > 30 g/m) as well as extremely high powder feeding (F/V > 35 g/m).

#### 3.1.3. Clad Height

**Figure 7:**
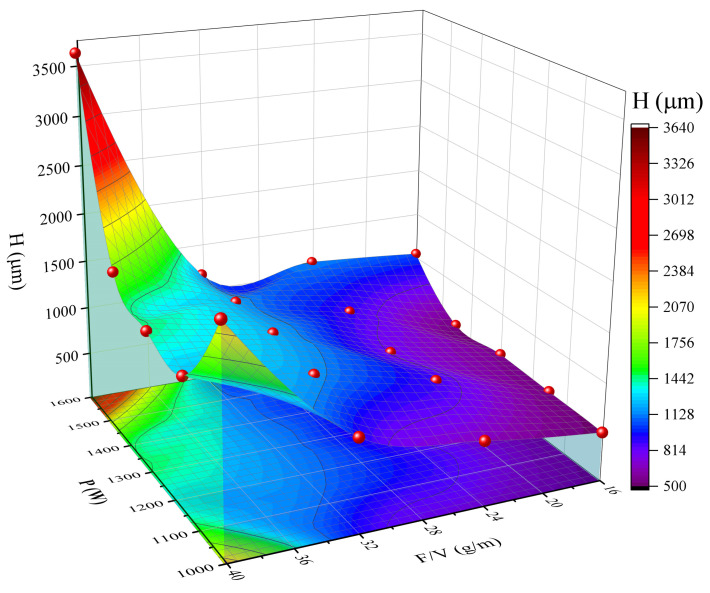
Surface plot of height, laser power, powder feeding rate/scanning speed (F/V).

Figure 7 illustrates that the formation mechanism of cladding height (H) exhibits a complex response to both feed rate to velocity ratio (F/V) and power (P). A single-factor effect analysis indicates that a 10 g/m increase in F/V results in a linear increase in H of approximately 300 μm, which directly correlates with an increase in powder deposition rate per unit time. The power parameter demonstrates a significant threshold effect: when P is less than or equal to 1150 W, the energy input merely meets the basic cladding requirements, and H is predominantly controlled by F/V. Conversely, when P exceeds the threshold of 1300 W, a dynamic equilibrium is achieved between the energy of the melt pool and powder deposition, leading to an increase in the H growth rate of approximately 40%. Experimental data corroborate this threshold effect, as evidenced by the comparison between sample No. 16 (P = 1450 W, F/V = 32.13 g/m) and sample No. 12 (P = 1300 W, F/V = 21.42 g/m).

Process matching analysis indicates that when the power (P) is equal to or greater than 1300 W and the feed rate to velocity ratio (F/V) is equal to or greater than 30 g/m, the height (H) stabilizes within the range of 1000–1400 μm. This range corresponds to a high-quality forming window characterized by stable melt pool flow. It is crucial to exercise caution with excessive power (P > 1500 W), as this can lead to over-melting and evaporation of the powder, resulting in an abnormal decrease in H. A graded control strategy is recommended: prioritize adjusting the F/V ratio (28–35 g/m) for precise control of H under normal conditions. When high deposition requirements are necessary, the power should be simultaneously increased to the range of 1300–1450 W.

#### 3.1.4. Dilution Rate

**Figure 8:**
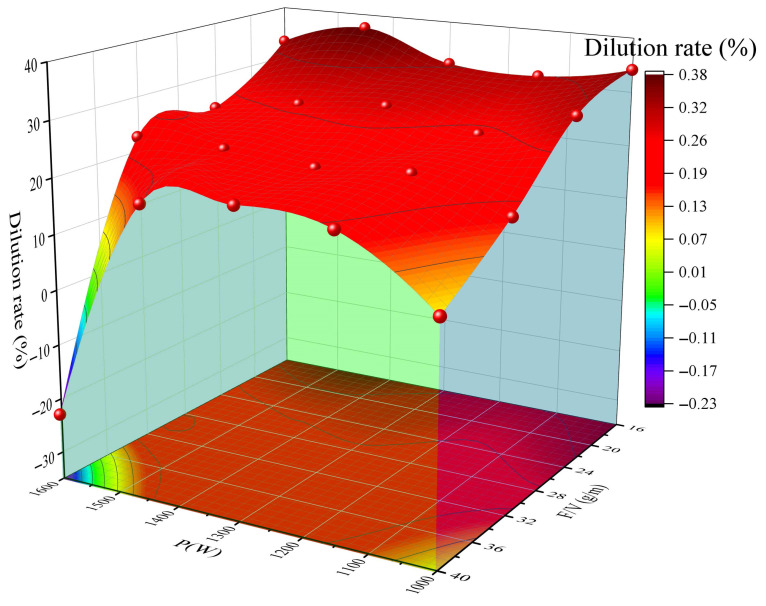
Surface plot of dilution rate, laser power, and powder feeding rate/scanning speed (F/V).

Figure 8 illustrates the evolution of the dilution rate (D), highlighting the trade-off between energy density and powder deposition. In the high-temperature region (P > 1300 W, F/V 28 g/m^2^ range, the cooling effect of powder feeding can offset the power increase of approximately 150 W (for example, at point 16, where P = 1450 W, F/V = 32.13 g/m^2^, D = 22.55%). Experimental results demonstrate that the parameter combination of P = 1200 ± 50 W and F/V = 28–32 g/m^2^ can achieve a dilution rate of D = 20–25%.

#### 3.1.5. Element Deviation

**Figure 9:**
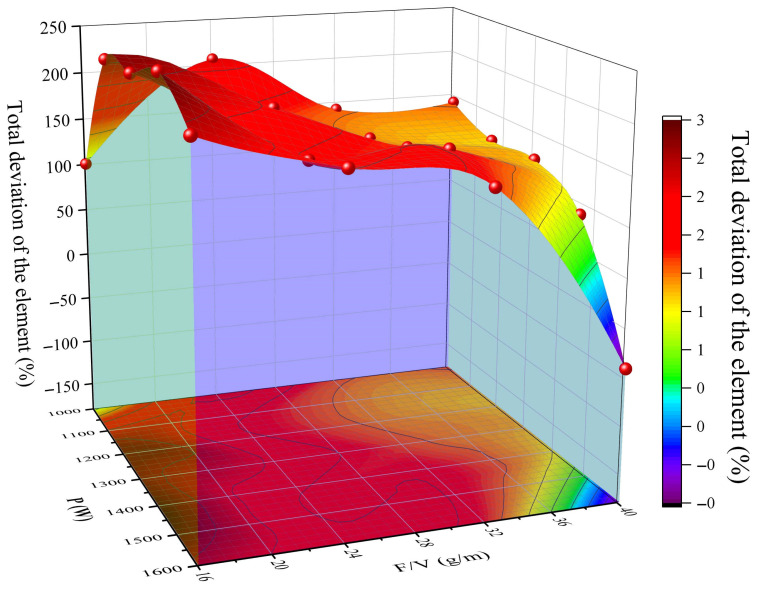
Surface plot of element deviation vs. laser power, powder feed rate/scanning speed (F/V).

As illustrated in Figure 9, the effects of laser power (P) and F/V ratio (powder feed rate/scanning speed) on the total elemental deviation and dilution rate in HEA exhibit a significant synergistic effect. The optimal range for laser power is between 1150 and 1300 W, with an F/V ratio of 30–40 g/m (corresponding to a powder feed rate of 0.28–0.32 g/s and a scanning speed of 8–10 mm/s), which reduces the total elemental deviation to between 118% and 143%. The lowest deviation occurs at the parameter set of 1150 W, 0.32 g/s, and 8 mm/s. Additionally, the dilution rate is maintained at 18.77%. Low F/V ratios (less than 25 g/m) result in excessive matrix dilution due to inadequate powder feeding; for instance, at F/V = 17.5 g/m, the deviation reaches 214%. Conversely, high laser powers (greater than 1300 W) or elevated scanning speeds (exceeding 12 mm/s) lead to unbalanced heat input, exacerbating elemental segregation. Element-specific deviations are primarily attributed to the interplay of differences in physical properties and matrix dilution. Aluminum, owing to its low melting point (660 °C), tends to evaporate from the upper layer of the melt pool, resulting in a substantial negative deviation of −11.18% in the middle layer. The lower deviation observed for chromium and iron is a consequence of matrix dilution, as 430 stainless steel comprises 17% chromium and 83% iron. An increased dilution rate (e.g., 36.96% dilution in sample No. 1) raises the Fe/Cr ratio in the cladding layer, leading to measured values that are more closely aligned with the matrix composition, with the chromium deviation decreasing from a theoretical 20% to 18.3%. For aluminum, cobalt, and nickel, deviations are predominantly influenced by evaporation or segregation due to the absence of corresponding elements in the matrix. Effective optimization necessitates a balance between parameter matching and compensation for physical properties. The combination of 1150 W laser power, 0.32 g/s powder feed, and 8 mm/s scanning speed concurrently achieves a low deviation of 118%, a controllable dilution rate of 18.77%, and mitigates aluminum evaporation. Furthermore, the use of argon shielding effectively reduces oxidation losses.

#### 3.1.6. Hardness

**Figure 10:**
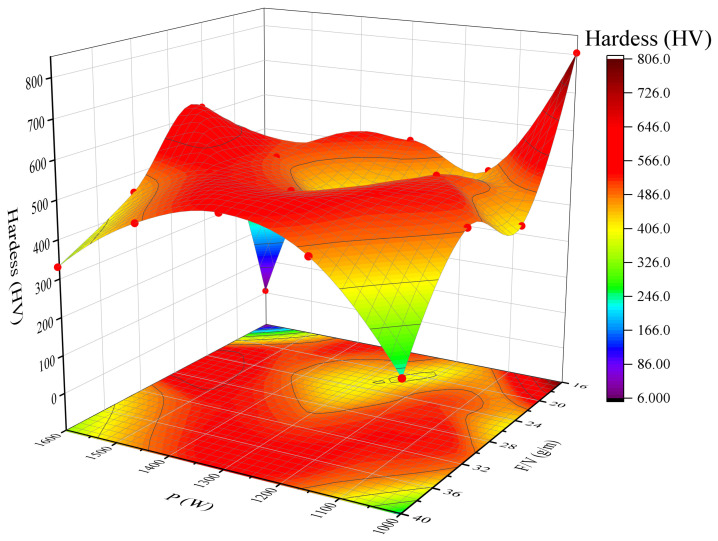
Surface plot of hardness vs. laser power, powder feeding rate/scanning speed (F/V).

As shown in Figure 10, the nonlinear relationship between hardness, laser power (P), and the F/V ratio primarily arises from the dynamic interplay between the cladding layer density and the matrix dilution rate. At high laser power (1300 W) and medium-to-high F/V values (36.1 g/m), the molten pool undergoes complete melting (with 0% porosity), while moderate dilution (approximately 21%) preserves the fine grain strengthening effect of the HEA, resulting in a peak hardness of 602 HV.In the lower power range (1000 W), hardness fluctuates between 365 and 556 HV due to an imbalance between energy input and powder feeding. When F/V > 25 g/m, residual unmelted particles reduce the overall density. On the other hand, when F/V < 25 g/m, excessive mixing of matrix Fe/Cr (resulting in a dilution rate > 30%) leads to a reduction in the hard phase content. When P is between 1150 W and 1300 W, and F/V falls within the range of 25–35 g/m, the energy input aligns with the requirements for powder melting, stabilizing the dilution rate at 20–25%. Solution strengthening becomes the dominant mechanism, and the hardness stabilizes between 500 and 600 HV. Outside of this range, excessive laser power causes grain coarsening, while F/V < 25 g/m leads to increased matrix dilution, significantly reducing hardness by 20–30%. To balance both hardness and process stability, a laser power range of 1150–1300 W and an F/V ratio of 28–32 g/m are recommended to achieve a hardness between 500 and 550 HV.

### 3.2. Signal-to-Noise Ratio Analysis and Variance Analysis of Taguchi Method

#### 3.2.1. Signal-to-Noise Ratio Analysis

In the Taguchi method, experimental results are transformed into the signal-to-noise ratio (S/N) format. These results can be categorized into three types based on the research objectives: long-term characteristics (LTB), short-term characteristics (STB), and non-target characteristics (NTB) [28].Equation (4) presents the formula for calculating the signal-to-noise ratio. In this study, the response targets included cladding width (W), cladding height (H), dilution rate (η), element deviation rate (α), and hardness (φ). To minimize defects associated with single-pass overlap deposition, a target single-pass width of 3 mm and a height of 1.2 mm were established. To mitigate energy waste resulting from suboptimal metallurgical combinations due to excessively high or low dilution rates, a target dilution rate of 20% was chosen. It is preferable for the various elements of the cladding layer to approach their ideal values; therefore, a smaller element deviation rate is more desirable. Increased hardness can elevate the internal stress within the cladding layer, potentially leading to cracking, whereas reduced hardness may compromise the wear resistance of the cladding layer. Consequently, a target hardness of 550 HV was selected. The calculated signal-to-noise ratios for the response targets of width, height, dilution rate, element deviation rate, and hardness are summarized in Table 6.

(4) $$S/N=\left\{\begin{array}{ll} -10\log\left(\frac{1}{n}\sum_{i=1}^{n}\frac{1}{y_{i}^{2}}\right) & LTB\\ -10\log\left(\frac{1}{n}\sum_{i=1}^{n}y_{i}^{2}\right) & STB\\ -10\log\left(\frac{1}{n}\sum_{i=1}^{n}{\left(y_{i}-m\right)}^{2}\right) & NTB \end{array}\right.$$

**Figure 11:**
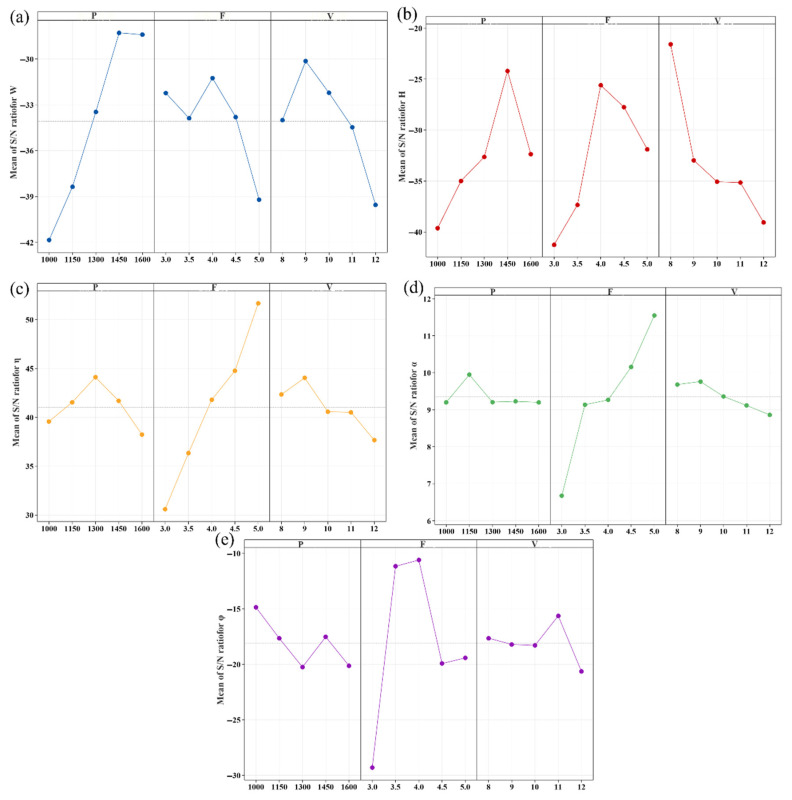
Signal-to-noise ratio responses for different response targets: (**a**) SNR main effect plot for W, (**b**) SNR main effect plot for H, (**c**) SNR main effect plot for η, (**d**) SNR main effect plot for α, and (**e**) SNR main effect plot for φ.

Figure 11 shows the main effect plots of the signal-to-noise ratio for the width, height, dilution rate, element deviation rate, and hardness of a single-pass laser-clad AlCoCrFeNi HEA coating. Figure 11a shows that the signal-to-noise ratio of the cladding width increases with increasing laser power. However, the cladding height, dilution rate, element deviation rate, and hardness are most affected by the powder feed rate, as shown in Figure 11b–e.

#### 3.2.2. Analysis of Variance

Variance analysis can be employed to investigate the parameters that significantly influence geometric characteristics [29]. Minitab software (Minitab^®^ 21.1, 64-bit) facilitates the generation of a signal-to-noise ratio response table and a corresponding response graph. The signal-to-noise ratio response table indicates which factors exert a greater influence on the response target, categorizing their impacts as large, small, or negligible. Furthermore, the optimal process parameter can be identified from the signal-to-noise ratio response graph, which displays the highest value.

Variance analysis is conducted by summing the squared deviations from the overall mean signal-to-noise ratio. In this experiment, variance analysis was employed to evaluate the contribution of each process parameter—width, height, dilution rate, element deviation rate, and hardness—allowing for the identification of the process parameter with the highest contribution rate. The Fisher value (F) for each process parameter was calculated. Some scholars [30] have proposed that if F > 4 and the *p* value is less than 0.05, it indicates that the process parameter has a significant impact.

Table 7 presents the variance results for laser cladding width (W), height (H), dilution rate (η), element deviation rate (α), and hardness (φ). As illustrated in Table 7, laser power exerts the most significant influence on single-pass width, with a contribution rate of 67.1%. This finding suggests that increasing laser power enhances the single-pass width. In contrast, scanning speed and powder feed speed are secondary factors, contributing 17.7% and 15.2%, respectively. Notably, powder feed speed has the most substantial effect on single-pass height, dilution rate, element deviation rate, and hardness, corroborating the earlier signal-to-noise ratio analysis, with contribution rates of 70.6%, 96.2%, 92.8%, and 82.5%, respectively. This indicates that increasing powder feed speed and the quantity of powder melted in a single pass promotes single-pass height, dilution rate, element deviation rate, and hardness.

### 3.3. Multi-Objective Optimization Based on GRA

The optimization of process parameters using the Taguchi method is effective for single-objective optimization; however, it is inadequate for scenarios involving multiple objectives. To address complex multi-response problems, a combination of the Taguchi method and the GRA can be employed [31,32,33]. This approach transforms the challenge of optimizing laser process parameters into the task of optimizing the grey relational degree, ultimately yielding the optimal parameter combination of the appropriate aspect ratio and dilution rate. The GRA consists of three steps: normalization of the signal-to-noise ratio, calculation of the grey relational coefficient, and determination of the grey relational degree [34].

First, due to the multi-scale problem of different response values, the experimental data needs to be normalized to between 0 and 1. The normalization formula is shown in (5)

(5) $$N_{ij}=\left\{\begin{array}{ll} \frac{SN_{ij}-\min(SN_{ij})}{\max(SN_{ij})-\min(SN_{ij})} & \;LTB\\ \frac{\max(SN_{ij})-SN_{ij}}{\max(SN_{ij})-\min(SN_{ij})} & \;STB \end{array}\right.$$

where *N_ij_* is the normalized value of the i-th trial, and *SN_ij_* is the SNR value of the i-th trial.

Then, the grey relational coefficients of the four process parameters can be obtained from Formula (6), which represents the relationship between the actual normalized signal-to-noise ratio and the ideal value.

(6) $$\varepsilon_{\mathrm{ij}}=\frac{\Delta_{\min}+\tau \Delta_{\max}}{\Delta_{\mathrm{ij}}+\tau \Delta_{\max}}$$

$\Delta_{ij}=\left|N_{0j}-N_{ij}\right|$ is the deviation sequence, N_0j_ is the j-th response value, $\Delta_{min}=min\{\Delta_{ij}\},\Delta_{max}=max\{\Delta_{ij}\}$, *τ*: discrimination coefficient, *τ* ∈ (0, 1), the value of this paper is 0.5.

Finally, the GRG considering the SNR combination of two response targets (Z, η) can be calculated by Formula (7).

(7) $$GRG_{i}=\frac{1}{n}\sum_{K=1}^{n}w_{k}\cdot \varepsilon_{i}(k)$$

where *n* is the number of response targets, and *w_k_* is the normalized weight value, which is assumed to be 1 in this paper.

Table 8 presents the grey relational coefficients and grey relational degrees for each response variable across the 25 experimental groups. To identify the laser cladding process parameters that yield optimal overall performance, the Taguchi method was integrated with grey relational analysis. As illustrated in the table, the highest grey relational degree was obtained for sample #16, which employed the process parameters P4F4V1 (laser power of 1450 W, powder feed rate of 4 r/min, and scanning speed of 8 mm/min).

**Figure 12:**
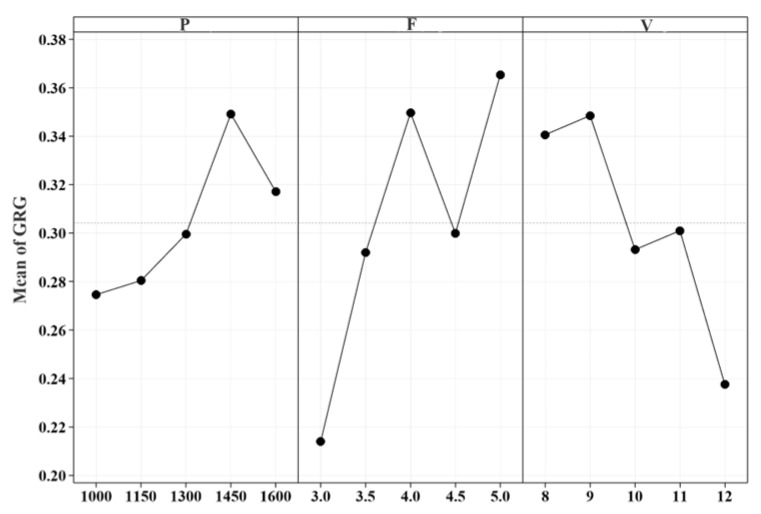
Main effect diagram of GRG.

Figure 12 illustrates the main effect diagram of the grey relational degree, while Table 9 presents the response table of the grey relational degree signal-to-noise ratio. The analysis reveals that the influence of the three process parameters on the grey relational degree ranks from largest to smallest as follows: powder feed rate, scanning speed, and laser power. This indicates that the powder feed rate is the most critical factor affecting the comprehensive performance of the cladding layer, followed by scanning speed, with laser power exerting the least influence.

**Figure 13:**
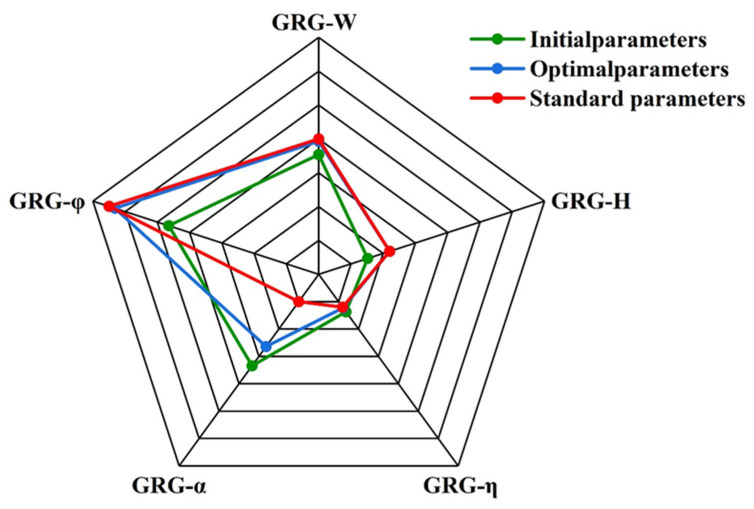
Radar chart of response under initial parameters P1F1V1 (#1), optimal parameters P4F4V1, and standard parameters.

To validate the effectiveness of laser cladding parameter optimization, this paper conducts a comparative analysis of samples obtained under the initial parameter group (P1F1V1, Group 1), the optimized parameter group (P4F4V1, Group 16), and a set of standard conventional parameters. By combining the response data for each group presented in Table 10 with the radar plot shown in Figure 13, the results indicate that the optimized parameter group demonstrates superior cladding layer geometry quality, characterized by moderate width and height, clear contours, a low dilution rate, and good forming continuity. In contrast, the initial group exhibited a wide but inadequately high cladding layer, likely due to insufficient powder melting resulting from low laser power. Although the standard group displayed moderate width, it also showed a high dilution rate, indicative of excessive substrate melting. The radar plot further illustrates the balanced performance of the optimized group in terms of width, height, and dilution rate, culminating in an overall geometrical profile that is closer to the ideal. In terms of elemental segregation, the optimized group demonstrated the most uniform distribution of major elements, including Al, Cr, and Fe, with minimal segregation. This finding indicates that the optimized parameters enhanced the diffusion and solidification of alloying elements within the melt pool, thereby promoting the formation of a fine and stable microstructure. In contrast, both the initial and standard groups exhibited significant segregation, which could potentially lead to localized performance degradation. Regarding hardness, the average microhardness of the optimized group was significantly greater than that of the other two groups, by approximately 15%. This enhancement can be attributed to the optimal matching of laser heat input and powder supply, which facilitated microstructure densification and grain refinement. The hardness index depicted in Figure 13 is positioned at the outermost edge of the radar plot, further underscoring the superior mechanical properties of the optimized group. In summary, the optimized parameter P4F4V1 surpassed the initial and standard parameters across multiple key response metrics, including geometric formability, compositional uniformity, and microhardness. This conclusively validates the effectiveness of the Taguchi method and grey relational analysis in multi-index optimization and offers a feasible and reliable process reference for the fabrication of high-performance AlCoCrFeNi HEA laser cladding layers.

## 4. Conclusions

This paper investigates the laser cladding single-pass coating of the AlCoCrFeNi HEA. It explores the influence of process parameters on single-pass geometric formation, element distribution, and mechanical properties. Furthermore, it achieves multi-objective optimization by integrating the Taguchi method with GRA.

(1) Based on Taguchi orthogonal design and GRA, this study systematically reveals the influence of laser power, scanning speed, and powder feeding rate on the geometric formation, elemental segregation, and mechanical properties of AlCoCrFeNi HEA coatings. Notably, laser power exerts the most significant effect on the width of the cladding layer, while the powder feeding rate predominantly influences the height, dilution rate, elemental deviation rate, and hardness.
(2) The Taguchi signal-to-noise ratio and variance analysis indicate that the cladding width is predominantly influenced by laser power, which accounts for a contribution rate of up to 67.1%. In contrast, the coating height, dilution rate, element deviation rate, and hardness are primarily influenced by the powder feed rate, with contributions exceeding 70%. These findings underscore the critical regulatory role of the powder deposition process in determining the overall performance of the cladding layer.
(3) Through multi-objective optimization combined with grey relational analysis, the optimal process parameter combination P4F4V1 was identified, consisting of a laser power of 1450 W, a powder feed rate of 4 r/min, and a scanning speed of 480 mm/min. Verification experiments demonstrated that this combination exhibited superior performance in terms of coating build quality, elemental uniformity, and microhardness. Compared to the initial parameters, significant improvements were observed in the dilution rate and elemental segregation issues, resulting in an approximate 15% increase in hardness.
(4) This study not only validates the effectiveness of the Taguchi-Grey Relational Analysis for optimizing complex multi-index problems but also offers a reliable methodology for the process design of HEA laser cladding coatings. The findings provide technical support for surface strengthening and life extension of critical components, such as aerospace engine turbine discs, under extreme operating conditions. Furthermore, they hold significant implications for the application and promotion of HEA across aerospace and other industries.

## Figures and Tables

**Table 1:** Details of powders used for deposition.

S. No.	Element	Make	Purity	Particle Size
1	Al	Changsha Tianjiu Metal Materials Co., Changsha, China	99.9%	45–106 μm
2	Co	Changsha Tianjiu Metal Materials Co., Changsha, China	99.9%	45–106 μm
3	Cr	Changsha Tianjiu Metal Materials Co., Changsha, China	99.9%	15–53 μm
4	Fe	Changsha Tianjiu Metal Materials Co., Changsha, China	99.9%	45–106 μm
5	Ni	Changsha Tianjiu Metal Materials Co., Changsha, China	99.9%	15–53 μm

**Table 2:** Chemical Composition of 430 Stainless Steel (wt%).

C	Si	Mn	Ni	Cr	Fe
≤0.12	≤0.75	≤1	≤0.6	16~18	Bal.

**Table 3:** Single-track experimental parameters.

Level	1	2	3	4	5
P/W	1000	1150	1300	1450	1600
V/mm·min^−1^	480	540	600	660	720
F/r·min^−1^	3	3.5	4	4.5	5

**Table 4:** L_25_ Taguchi orthogonal experimental design.

NO.	P (W)	V (mm/min)	F (r/min)	NO.	P (W)	V (mm/min)	F (r/min)
1	1000	480	3	14	1300	660	5
2	1000	540	4	15	1300	720	3.5
3	1000	600	5	16	1450	480	4
4	1000	660	3.5	17	1450	540	5
5	1000	720	4.5	18	1450	600	3.5
6	1150	480	5	19	1450	660	4.5
7	1150	540	3.5	20	1450	720	3
8	1150	600	4.5	21	1600	480	3.5
9	1150	660	3	22	1600	540	4.5
10	1150	720	4	23	1600	600	3
11	1300	480	4.5	24	1600	660	4
12	1300	540	3	25	1600	720	5
13	1300	600	4				

**Table 5:** Measured data of geometric parameters of a single-pass cladding layer.

No.	W (μm)	H (μm)	η (%)	α (%)	φ (HV)
1	2539.11	518.05	36.96%	234%	365
2	2498.21	514.56	22.64%	155%	553
3	2246.01	947.48	17.69%	142%	492
4	2368.70	637.33	32.43%	170%	556
5	2177.84	817.97	23.11%	179%	475
6	2539.11	1370.10	18.77%	118%	469
7	2661.81	903.17	23.84%	163%	519
8	2597.05	1049.73	23.11%	143%	535
9	2600.46	616.88	30.71%	214%	483
10	2515.25	800.93	28.21%	173%	519
11	2702.71	1288.30	21.97%	153%	602
12	2941.28	681.64	34.84%	222%	254
13	2787.91	923.62	22.41%	167%	534
14	2532.29	1121.30	20.52%	144%	524
15	2580.01	746.40	27.99%	192%	495
16	2931.05	1199.69	22.55%	164%	532
17	2770.87	1414.40	20.77%	135%	514
18	2954.91	835.01	28.69%	179%	517
19	2808.36	978.15	23.99%	153%	499
20	2729.97	548.72	37.47%	256%	479
21	3227.57	954.30	27.79%	171%	544
22	3046.93	1175.83	22.65%	151%	451
23	3121.91	719.13	34.85%	235%	296
24	3057.16	865.68	28.39%	206%	597
25	2467.54	1605.26	16.65%	125%	600

**Table 6:** Signal-to-noise ratio response table of W, H, η, α, and φ.

Cladding width (W)	Level	P	V	F
	1	−41.85	−34.00	−32.23
	2	−38.36	−30.14	−33.88
	3	−33.47	−32.22	−31.25
	4	−28.29	−34.47	−33.81
	5	−28.41	−39.55	−39.21
	Delta	13.56	9.41	7.96
	Sorting	1	2	3
Cladding height (H)	Level	P	V	F
	1	−39.64	−21.59	−41.26
	2	−35.01	−32.97	−37.35
	3	−32.64	−35.08	−25.60
	4	−24.20	−35.15	−27.76
	5	−32.37	−39.07	−31.89
	Delta	15.44	17.48	15.66
	Sorting	3	2	1
Dilution rate (η)	Level	P	V	F
	1	39.57	42.34	30.60
	2	41.54	44.04	36.33
	3	44.11	40.58	41.79
	4	41.69	40.52	44.77
	5	38.24	37.67	51.66
	Delta	5.87	6.37	21.06
	Sorting	3	2	1
Element Deviation (α)	Level	P	V	F
	1	9.19	9.68	6.67
	2	9.94	9.76	9.13
	3	9.20	9.35	9.26
	4	9.22	9.11	10.15
	5	9.20	8.86	11.54
	Delta	0.74	0.90	4.87
	Sorting	3	2	1
Hardness (φ)	Level	P	V	F
	1	−14.88	−17.65	−29.30
	2	−17.65	−18.22	−11.18
	3	−20.25	−18.30	−10.62
	4	−17.54	−15.64	−19.93
	5	−20.14	−20.65	−19.43
	Delta	5.38	5.01	18.68
	Sorting	2	3	1

**Table 7:** Variance results of cladding width (W), cladding height (H), dilution rate (η), element deviation rate (α) and hardness (φ).

Response Target	Level	Laser Power (A)	Scan Speed (B)	Powder Feed Rate (C)	Deviation	Total
Cladding width (W)	Degrees of freedom	4	4	4	12	24
	Adj SS	1,127,204	297,505	254,904	59,926	1,739,539
	Adj MS	281,801	74,376	63,726	4994	
	F-number	56.43	14.89	12.76		
	*p*-value	0.000	0.000	0.000		
	Contribution rate (%)	67.1	17.7	15.2		
Cladding height (H)	Degrees of freedom	4	4	4	12	24
	Adj SS	410,255	139,541	1,321,199	183,347	2,054,342
	Adj MS	102,564	34,885	330,300	15,279	
	F-number	6.71	2.28	21.62		
	*p*-value	0.004	0.120	0.000		
	Contribution rate (%)	21.9	7.5	70.6		
Dilution rate (η)	Degrees of freedom	4	4	4	12	24
	Adj SS	0.001071	0.001805	0.073138	0.009176	0.085190
	Adj MS	0.000268	0.000451	0.018285	0.000765	
	F-number	0.35	0.59	23.91		
	*p*-value	0.839	0.676	0.000		
	Contribution rate (%)	1.4	2.4	96.2		
Element deviation rate (α)	Degrees of freedom	4	4	4	12	24
	Adj SS	0.08527	0.12483	2.72517	0.21841	3.15368
	Adj MS	0.02132	0.03121	0.68129	0.01820	
	F-number	1.17	1.71	37.43		
	*p*-value	0.372	0.211	0.000		
	Contribution rate (%)	2.9	4.3	92.8		
Hardness (φ)	Degrees of freedom	4	4	4	12	24
	Adj SS	2501	17,447	94,238	53,182	167,367
	Adj MS	625.3	4361.6	23,559.5	4431.8	
	F-number	0.14	0.98	5.32		
	*p*-value	0.964	0.452	0.011		
	Contribution rate (%)	2.2	15.3	82.5		

**Table 8:** GRA table of response targets.

Serial Number	Grey Relational Coefficient					Grey Relational Degree	
	W	H	η	α	φ	Digital	Sorting
1	0.199871	0.166759	0.167866	0.184298	0.182566	0.180271888	25
2	0.194188	0.166667	0.302078	0.365285	1	0.405643592	5
3	0.170913	0.186857	0.320448	0.456337	0.2388862	0.274688269	16
4	0.18034	0.170304	0.18132	0.297478	0.6099888	0.287886168	13
5	0.166667	0.177921	0.282405	0.271582	0.2235406	0.224423068	20
6	0.199871	0.196265	0.450788	1	0.219368	0.413258282	4
7	0.223707	0.183263	0.260181	0.326363	0.2863394	0.25597062	18
8	0.209562	0.199414	0.282106	0.442867	0.3725587	0.301301646	11
9	0.210206	0.169634	0.188547	0.207304	0.2299539	0.201129065	23
10	0.196457	0.177029	0.20303	0.288141	0.2874218	0.230415781	19
11	0.235397	0.214156	0.345705	0.3767	0.2464071	0.283673074	14
12	0.687349	0.171862	0.173399	0.196966	0.1666667	0.279248601	15
13	0.272735	0.184833	0.314634	0.308534	0.3641535	0.288977808	12
14	0.198865	0.217639	1	0.441112	0.3029431	0.432111891	2
15	0.206475	0.17446	0.204647	0.242015	0.2420661	0.213932776	22
16	0.57755	1	0.306987	0.320678	0.3488185	0.510806663	1
17	0.263182	0.190642	0.644903	0.541307	0.2737748	0.382761688	7
18	1	0.178864	0.199809	0.27245	0.2829375	0.386812055	6
19	0.286374	0.189839	0.256581	0.374614	0.2478661	0.271054908	17
20	0.244948	0.167594	0.166667	0.166667	0.2273288	0.194640689	24
21	0.264	0.18748	0.206177	0.293766	0.6244996	0.315184516	9
22	0.199871	0.166759	0.167866	0.184298	0.182566	0.180271888	25
23	0.194188	0.166667	0.302078	0.365285	1	0.405643592	5
24	0.170913	0.186857	0.320448	0.456337	0.2388862	0.274688269	16
25	0.18034	0.170304	0.18132	0.297478	0.6099888	0.287886168	13

**Table 9:** GRG average response table.

Level	P	V	F
1	0.2746	0.3406	0.2139
2	0.2804	0.3485	0.2920
3	0.2996	0.2932	0.3497
4	0.3492	0.3010	0.2999
5	0.3171	0.2375	0.3654
Delta	0.0746	0.1110	0.1515
Sorting	3	2	1

**Table 10:** Response of samples under optimal parameters and initial parameters.

Compare Projects	Initial Parameters	Optimal Parameters	Target
Parameter combination	P1F1V1	P4F4V1	
W (mm)	2.539	2.931	Looking
H (mm)	0.518	1.200	Looking
η (%)	36.96%	22.55	Looking
α (%)	234%	164%	Hope
φ (HV)	365	532	Looking

## Data Availability

The original contributions presented in this study are included in the article. Further inquiries can be directed to the corresponding author.

## References

[1] Backman D.G., Williams J.C. (1992). Advanced Materials for Aircraft Engine Applications. Science.

[2] Miller S. (1996). Advanced materials mean advanced engines. Interdiscip. Sci. Rev..

[3] Jafari S., Nikolaidis T. (2018). Thermal Management Systems for Civil Aircraft Engines: Review, Challenges and Exploring the Future. Appl. Sci..

[4] Yan J.J., Yan G., Chen H.Y., Liu Z.Y., Yang L., Zhou Y.C. (2022). Real-time detection of damage evolution and failure of EB-PVD thermal barrier coatings using an environmental simulator with high-temperature and high-speed rotation. Surf. Coat. Technol..

[5] Everitt S. (2012). Developments in Advanced High Temperature Disc and Blade Materials for Aero-Engine Gas Turbine Applications. Ph.D. Thesis.

[6] Alnaeli M., Alnajideen M., Navaratne R., Shi H., Czyzewski P., Wang P., Eckart S., Alsaegh A., Alnasif A., Mashruk S. (2023). High-Temperature Materials for Complex Components in Ammonia/Hydrogen Gas Turbines: A Critical Review. Energies.

[7] DeMasi-Marcin J.T., Gupta D.K. (1994). Protective coatings in the gas turbine engine. Surf. Coat. Technol..

[8] Peng Z., Han A., Wang C., Jin H., Zhang X. (2024). Ultrasonic vibration cutting of advanced aerospace materials: A critical review of in-service functional performance. J. Intell. Manuf. Spec. Equip..

[9] McLEAN A.F., Hartsock D.L., Wachtman J.B. (1989). 2—Design with Structural Ceramics Treatise on Materials Science & Technology Structural Ceramics.

[10] Akande I.G., Oluwole O.O., Fayomi O.S.I., Odunlami O.A. (2021). Overview of mechanical, microstructural, oxidation properties and high-temperature applications of superalloys. Mater. Today Proc..

[11] Yuan W., Li R., Chen Z., Gu J., Tian Y. (2021). A comparative study on microstructure and properties of traditional laser cladding and high-speed laser cladding of Ni45 alloy coatings. Surf. Coat. Technol..

[12] Wang K., Liu W., Hong Y., Sohan H.M.S., Tong Y., Hu Y., Zhang M., Zhang J., Xiang D., Fu H. (2023). An Overview of Technological Parameter Optimization in the Case of Laser Cladding. Coatings.

[13] Prakash O., Chandrakar R.L.M., Verma J., Kumar A., Jaiswal A. (2024). Laser cladding technology for high entropy alloys: Effect and applications. Mater. Res. Express.

[14] Li Y., Zhou S., Zhang Y., Brechtl J., Liaw P.K. (2021). Future Research Directions and Applications for High-Entropy Materials. High-Entropy Materials: Theory, Experiments, and Applications.

[15] Wang X., Liu Q., Wang X. (2025). High-Entropy Materials: From Bulk to Sub-nano. Adv. Funct. Mater..

[16] Zhang C., Zhu J., Zheng H., Li H., Liu S., Cheng G.J. (2020). A review on microstructures and properties of high entropy alloys manufactured by selective laser melting. Int. J. Extrem. Manuf..

[17] Brechtl J., Chen S., Lee C., Shi Y., Feng R., Xie X., Hamblin D., Coleman A.M., Straka B., Shortt H. (2020). A Review of the Serrated-Flow Phenomenon and Its Role in the Deformation Behavior of High-Entropy Alloys. Metals.

[18] Lin C., Yao Y. (2023). Corrosion-Resistant Coating Based on High-Entropy Alloys. Metals.

[19] Yu J., Mu R. Research Status of High Entropy Thermal Barrier Coatings: A Review. Proceedings of the 2021 3rd International Conference on Artificial Intelligence and Advanced Manufacture (AIAM).

[20] Li B., Sun J., Li X., Zhao J. (2024). Mechanical behavior of high-entropy intermetallic compounds and high-entropy ceramics. J. Mater. Chem. A.

[21] Lu J., Chen Y., Li L., Zhang H., Zhang X., Zhao X. (2023). An in-situ oxide-dispersion-strengthened AlCoCrFeNiY high-entropy alloy composite coating prepared by AC-HVAF with superior oxidation and spallation resistance. Compos. Part B Eng..

[22] Liu H., Liu J., Chen P., Yang H. (2019). Microstructure and high temperature wear behaviour of in-situ TiC reinforced AlCoCrFeNi-based high-entropy alloy composite coatings fabricated by laser cladding. Opt. Laser Technol..

[23] Odabas O., Karaoglanli A.C., Ozgurluk Y., Binal G. (2025). Evaluation of high temperature oxidation resistance of AlCoCrFeNiZr high-entropy alloy (HEA) coating system at 1000 °C and 1100 °C. Surf. Coat. Technol..

[24] Joseph J., Haghdadi N., Shamlaye K., Hodgson P., Barnett M., Fabijanic D. (2019). The sliding wear behaviour of CoCrFeMnNi and AlxCoCrFeNi high entropy alloys at elevated temperatures. Wear.

[25] Zhang L., Ye Q., Ji Y., Wang Y., Yang B. (2025). Hot corrosion resistance of AlCoCrFeNi2. 1 coatings at 900 °C. J. Mater. Res. Technol..

[26] Komvopoulos K., Nagarathnam K. (1990). Processing and Characterization of Laser-Cladded Coating Materials. J. Eng. Mater. Technol..

[27] Wang K., Zhang Z., Xiang D., Ju J. (2022). Research and Progress of Laser Cladding: Process, Materials and Applications. Coatings.

[28] Xiao W., Liu Y., Huang J., Zou S., Ren Z., Liu S., Wang Y. (2025). Process parameter optimization for laser directed energy deposition B4C/Al neutron absorbing material via Taguchi method. Opt. Laser Technol..

[29] Qasim A., Nisar S., Shah A., Khalid M.S., Sheikh M.A. (2015). Optimization of process parameters for machining of AISI-1045 steel using Taguchi design and ANOVA. Simul. Model. Pract. Theory.

[30] Meng F., Yang J. (2023). Process capability analysis of Taguchi index Cpm based on generalized p-value. Qual. Reliab. Eng. Int..

[31] Kuo Y., Yang T., Huang G.-W. (2008). The use of a grey-based Taguchi method for optimizing multi-response simulation problems. Eng. Optim..

[32] Al-Refaie A. (2010). Grey-data envelopment analysis approach for solving the multi-response problem in the Taguchi method. Proc. Inst. Mech. Eng. Part B J. Eng. Manuf..

[33] Li N., Chen Y.-J., Kong D.-D. (2019). Multi-response optimization of Ti-6Al-4V turning operations using Taguchi-based grey relational analysis coupled with kernel principal component analysis. Adv. Manuf..

[34] Lu H.S., Chang C.K., Hwang N.C., Chung C.T. (2009). Grey relational analysis coupled with principal component analysis for optimization design of the cutting parameters in high-speed end milling. J. Mater. Process. Technol..

